# Osteoarthritis: epidemiology, risk factors, molecular pathogenesis, therapeutic progress, and future perspectives

**DOI:** 10.1186/s43556-026-00658-3

**Published:** 2026-11-13

**Authors:** Kai Huang, Haili Cai

**Affiliations:** 1 Tongde Hospital of Zhejiang Province, No.234 Gucui Road, Hangzhou, Zhejiang Province China; 2 Department of Ultrasound, The 903rd Hospital of the Joint Logistic Support Force, Hangzhou, China

**Keywords:** Osteoarthritis, Whole-joint pathogenesis, Lipid metabolism, Molecular endotypes, Disease-modifying therapies, Precision medicine

## Abstract

Osteoarthritis (OA) is a heterogeneous whole-joint disease and a leading cause of pain and disability worldwide. Its development and progression reflect complex interactions among ageing, genetic susceptibility, mechanical stress, inflammation, and metabolic dysfunction. This review integrates current knowledge of OA epidemiology, risk factors, molecular pathogenesis, and therapeutic advances, with particular emphasis on lipid metabolism as an emerging regulator of joint homeostasis. We introduce lipid metabolic coding as a conceptual framework describing how lipid-derived substrates and signals are processed in a context-dependent manner to influence cellular identity, stress responses, and inter-tissue communication. Altered fatty-acid metabolism, de novo lipogenesis, lipid-droplet turnover, cholesterol handling, and post-translational modifications may contribute to cartilage degeneration and whole-joint pathology. We further discuss four overlapping, potentially stage-dependent lipid-associated endotypes as testable models rather than established clinical classifications. Although experimental evidence identifies promising metabolic targets, their causal relevance and therapeutic utility in human OA remain incompletely defined. Integrating longitudinal clinical data with spatial multi-omics and functional validation may clarify these mechanisms and support the development of more precisely targeted, disease-modifying therapies.

## Introduction

Osteoarthritis (OA) is the most common form of arthritis and a leading cause of chronic pain, functional impairment, and loss of mobility worldwide. Its burden is increasing rapidly with population ageing and the rising prevalence of obesity and metabolic disorders. In 2020, approximately 595 million people were living with OA, corresponding to 7.6% of the global population, and the number of affected individuals is projected to increase substantially by 2050 [1]. Beyond its high prevalence, OA is characterized by marked heterogeneity in anatomical distribution, clinical manifestations, structural progression, and biological drivers [2, 3].

Historically, OA was regarded predominantly as a consequence of mechanical “wear and tear”, ageing, and progressive degradation of articular cartilage [4, 5]. OA is now increasingly recognized as a multifactorial disorder of the whole joint, involving coordinated pathological changes in articular cartilage, synovium, subchondral bone, menisci, ligaments, periarticular tissues, and the local immune and metabolic microenvironment [6–8]. Mechanical injury and abnormal loading remain central contributors, but they interact with ageing, genetic susceptibility, systemic inflammation, obesity, diabetes, dyslipidaemia, and other metabolic factors to influence disease initiation and progression [9–12]. Notably, the association between obesity and OA extends beyond weight-bearing joints, supporting the contribution of systemic biological mechanisms in addition to excessive mechanical loading [12]. Thus, OA pathogenesis is better viewed as the result of dynamic interactions among biomechanical, inflammatory, metabolic, and ageing-related processes. Patients with similar radiographic severity may differ substantially in the dominant molecular pathways operating within their joints, while the same risk factor may generate different pathological consequences depending on disease stage, joint site, sex, metabolic background, and local tissue responses. Such heterogeneity has stimulated increasing interest in moving beyond purely anatomical or radiographic descriptions toward mechanism-based phenotypes and endotypes. A comprehensive understanding of OA therefore requires integration of epidemiology and risk factors with whole-joint molecular pathology, cell-state remodeling, tissue crosstalk, and emerging therapeutic strategies.

Among the molecular processes that may connect systemic risk factors with local joint pathology, metabolic dysregulation has attracted particular attention. Lipids are especially relevant because they function not only as energy substrates and membrane components, but also as signaling molecules, inflammatory mediators, organelle stressors, and regulators of protein stability and chromatin states. Previous studies have implicated altered fatty acid uptake and oxidation, de novo lipogenesis, lipid-droplet turnover, cholesterol handling, and lipid-sensitive post-translational modifications in OA biology [13–19]. However, these processes are often considered as isolated metabolic abnormalities rather than as interconnected mechanisms through which lipid stress may influence persistent cellular states. We therefore retain lipid metabolic coding as a distinctive mechanistic lens in this review, referring to the context-dependent interpretation of lipid-derived substrates, metabolites, and stress signals by joint cells and their conversion into molecular and cellular outputs.

In this comprehensive review, we first summarize the epidemiology, clinical heterogeneity, and major risk factors of OA, followed by an integrated discussion of whole-joint molecular pathogenesis involving cartilage degeneration, synovial inflammation, subchondral bone remodeling, cellular stress, and major signaling networks. We then examine lipid metabolic coding in greater mechanistic depth, with particular attention to fatty acid flux, acetyl-CoA-dependent regulation, de novo lipogenesis, lipid-droplet dynamics, cholesterol handling, organelle stress, post-translational modifications, spatial remodeling, and candidate lipid-associated OA endotypes. Finally, we review current and emerging therapeutic approaches, including non-pharmacological, pharmacological, surgical, regenerative, metabolic, and precision lipid-directed strategies, and discuss the major challenges for translating molecular endotyping into future personalized OA management.

## Epidemiology and clinical heterogeneity of osteoarthritis

The epidemiology of OA is characterized not only by a large and rapidly increasing global burden, but also by substantial variation across age, sex, anatomical site, geographic region, and clinical phenotype. Instead, population-level patterns may reflect different combinations of biomechanical exposure, ageing, systemic metabolic risk, genetic susceptibility, and local joint responses. Understanding this heterogeneity provides an essential foundation for interpreting OA risk factors, molecular mechanisms, and therapeutic responses.

### Global burden and temporal trends

OA is among the most prevalent musculoskeletal disorders worldwide and is a major contributor to chronic pain, disability, and impaired mobility. According to the Global Burden of Disease Study 2021, approximately 595 million people were living with OA in 2020, corresponding to 7.6% of the global population and representing a 132.2% increase in the absolute number of cases since 1990 [1]. Among adults aged 30 years or older, approximately 14.8% were affected, indicating that OA is not confined to advanced old age but also imposes a substantial burden during working and early retirement years [1]. The associated disability burden has increased in parallel: the global number of years lived with disability attributable to OA more than doubled between 1990 and 2020, while the age-standardized rate also increased, indicating that population growth and ageing alone do not fully account for the expanding burden [1].

This upward trajectory is expected to continue. By 2050, the number of individuals affected by knee, hip, hand, and other forms of OA is projected to increase markedly, with estimated increases of approximately 49%−95% across anatomical sites compared with 2020. Knee OA is expected to remain the largest contributor, with more than 600 million individuals projected to be affected globally by 2050 [1]. These projections reflect the combined effects of population ageing, longer life expectancy, increasing obesity and metabolic comorbidity, previous joint injury, and other modifiable and non-modifiable risk exposures [2, 11]. Importantly, the growth in OA burden is unlikely to be evenly distributed. Regions undergoing rapid demographic transition and population expansion may experience particularly large increases in case numbers, emphasizing the need for prevention strategies that are adapted to local demographic and risk-factor profiles.

Pain, loss of physical function, reduced work participation, and the need for long-term medical or surgical care generate substantial individual and societal consequences. Moreover, because effective disease-modifying treatments remain limited, the increasing prevalence of OA is likely to place growing pressure on health-care systems unless prevention, early identification, and mechanism-based treatment can be improved.

### Age-, sex-, population-, and joint-specific patterns

Age is one of the strongest determinants of OA prevalence, but its effect is neither linear nor biologically uniform. Global prevalence rises steeply from middle age onward, with the highest rates observed in older adults^[[1]]^. Ageing increases cumulative mechanical exposure and is accompanied by changes in cartilage matrix integrity, cellular senescence, mitochondrial function, inflammatory regulation, and tissue repair capacity. Thus, chronological age should not be viewed simply as a measure of accumulated “wear”, but as a biological context that modifies the response of joint tissues to injury, inflammation, and metabolic stress.

Sex is another major source of epidemiological and clinical heterogeneity. Across global estimates, OA is more prevalent in women than in men, and the difference becomes more pronounced after midlife [20, 21]. Women account for approximately 60% of people living with OA worldwide and, in many cohorts, experience greater pain and functional limitation than men at comparable levels of structural disease [20]. However, sex differences are joint- and phenotype-dependent. Knee OA is generally more prevalent in women, whereas sex differences in hip OA vary according to whether OA is defined radiographically or symptomatically. Radiographic hip OA may be more frequent in men in some populations, whereas symptomatic hip OA is often more frequent in women [1, 22]. These observations indicate that sex-related differences cannot be explained by a single mechanism and may involve interactions among joint anatomy, alignment, muscle strength, body composition, hormonal status, genetic factors, pain processing, and health-care utilization.

These sex-related patterns are also relevant to the metabolic perspective developed later in this review. Sex hormones and body-composition differences can influence systemic lipid metabolism, adipose biology, inflammation, and mitochondrial function [23–26]; however, whether sex-specific hormonal environments directly modify lipid uptake, lipid flux, or lipid-sensitive signaling in human OA chondrocytes remains insufficiently established. This represents an important research gap and argues for systematic incorporation of sex as a biological variable in future metabolic and mechanistic studies of OA.

Anatomical site further contributes to disease heterogeneity. Knee OA is the most common form globally, followed by hand OA, whereas hip OA contributes a smaller proportion of overall prevalence. However, the relative contribution of different joint sites varies geographically. Knee OA predominates in most regions, while hand OA accounts for a larger proportion of cases in some populations, including parts of Central Asia and Eastern Europe [1]. Such population differences may reflect variation in age structure, obesity, occupational and recreational loading, previous injury, genetic background, and case ascertainment. They also caution against extrapolating epidemiological or mechanistic findings from one joint site or population directly to another.

### Clinical and molecular heterogeneity

The heterogeneity observed at the population level is mirrored clinically. Patients with OA can differ substantially in pain severity, functional impairment, rate of structural progression, affected joint tissues, comorbidities, and response to treatment, even when conventional radiographic severity appears similar [27, 28]. Conversely, substantial structural abnormalities may occur in individuals with relatively modest symptoms, illustrating the incomplete correspondence between radiographic damage and clinical disease. These observations have prompted increasing interest in moving beyond a single disease model toward stratification based on clinically observable phenotypes and biologically defined endotypes.

Phenotypes describe recognizable disease patterns based on features such as symptoms, imaging findings, anatomical site, obesity, inflammation, mechanical abnormalities, or pain characteristics. Endotypes aim to identify the underlying biological mechanisms responsible for these phenotypes and may therefore be more directly relevant to therapeutic selection [29]. Proposed OA endotypes include inflammatory, metabolic, biomechanical or injury-related, ageing/senescence-associated, bone-remodeling, and low-repair states, although their definitions vary across studies and substantial overlap is common [30, 31]. At present, no molecular endotype classification has been sufficiently standardized or prospectively validated for routine clinical decision-making. Nevertheless, defining mechanistically coherent patient subgroups is increasingly regarded as an important step toward targeted disease-modifying therapy.

Molecular and single-cell studies provide further support for this heterogeneity [32, 33]. OA cartilage does not contain a uniform population of progressively failing chondrocytes; instead, inflammatory, prehypertrophic, hypertrophic, fibrocartilage-like, and other disease-associated cell states have been identified, with their abundance and spatial distribution differing across tissue regions and disease contexts. However, cell-state heterogeneity should not be equated directly with clinically validated endotypes. Molecular states may coexist, overlap, or change during disease progression, and the dominant biological mechanism in an individual patient may vary with disease stage, joint compartment, systemic comorbidity, and treatment exposure.

A useful working model is therefore that OA contains shared core pathological processes, including matrix failure, inflammatory activation, abnormal tissue remodeling, and impaired repair, upon which patient-, joint-, and stage-specific biological programs are superimposed. It also provides the rationale for the subsequent sections of this review: first, to examine how major risk factors converge on whole-joint molecular pathology, and subsequently to consider lipid metabolic coding as one potential mechanistic layer within this broader, overlapping endotype architecture.

## Risk factors and disease initiation

OA develops through the interaction of intrinsic susceptibility and superimposed mechanical, metabolic, and inflammatory stress. Ageing, sex, and genetic background influence joint resilience, whereas injury, abnormal loading, obesity, diabetes, dyslipidaemia, and systemic inflammation can trigger or amplify disease processes. These factors rarely act independently and may converge on cartilage, synovium, and subchondral bone until adaptive responses become persistent pathological remodeling.

### Ageing, sex, and genetic susceptibility

Ageing is one of the strongest risk factors for OA, but its effect extends beyond cumulative “wear and tear”. Age-related joints show reduced matrix repair capacity, mitochondrial dysfunction, impaired proteostasis, and greater susceptibility to senescence-associated and inflammatory responses [34–36]. These changes reduce the ability of joint tissues to recover from mechanical or metabolic stress.

Sex modifies OA susceptibility through hormonal, biomechanical, and metabolic factors, although these effects vary by joint site [1]. Evidence for sex-specific lipid handling in human OA chondrocytes remains limited.

OA is additionally influenced by genetic susceptibility. It is a complex polygenic disorder in which variants affecting skeletal development, extracellular-matrix homeostasis, signaling, and tissue repair interact with ageing and environmental exposures. Such genetic heterogeneity may partly explain why similar external stresses lead to different disease trajectories.

### Mechanical loading, joint injury, and structural factors

Mechanical loading is essential for normal joint homeostasis; the relevant risk is therefore abnormal magnitude, distribution, duration, or recovery from loading. Malalignment, instability, meniscal dysfunction, altered bone morphology, muscle weakness, and repetitive occupational loading can redistribute forces across the articular surface and promote focal tissue stress [37, 38]. Joint injury is an especially important initiating event because ligament rupture, meniscal injury, and intra-articular trauma can produce long-lasting changes in joint mechanics and increase the risk of post-traumatic OA.

Mechanical injury also has biological consequences. Matrix disruption alters solute transport between synovial fluid, cartilage, and subchondral bone [39], while abnormal mechanical stress can modify chondrocyte metabolism and lipid handling. Mechanical stress has been reported to increase lipid-handling genes and impair microtubule-dependent lipid-droplet trafficking [13, 40]. Thus, mechanical stress can act as both a structural and metabolic driver.

### Obesity, metabolic syndrome, diabetes, and dyslipidaemia

Obesity is one of the most established modifiable risk factors for OA, particularly knee OA [2, 38]. Its effect cannot be explained solely by increased mechanical loading, because obesity is also associated with OA in non-weight-bearing joints [3, 11]. Adipose tissue releases free fatty acids, adipokines, cytokines, and other mediators that can influence cartilage, synovium, subchondral bone, and pain pathways [41–45].

Type 2 diabetes and other features of metabolic syndrome may further increase OA susceptibility through hyperglycaemia, oxidative stress, altered mitochondrial metabolism, and impaired inflammatory resolution [46]. Dyslipidaemia has also been associated with OA [47], but circulating lipid measures do not consistently predict cartilage loss, synovitis, pain, or structural progression [48]. This inconsistency suggests that serum lipid concentrations alone are insufficient to define lipid-associated OA. Local lipid composition and tissue-specific handling may be more informative, as reflected by changes in synovial-fluid lipid profiles [49, 50], cartilage lipidomics [51], fatty-acid metabolism [52], cholesterol efflux [53], and lipid-droplet turnover [40]. This distinction provides an important bridge to the lipid metabolic coding framework discussed later in this review.

### Inflammatory, lifestyle, and systemic factors

Low-grade inflammation acts as a cross-cutting amplifier of OA rather than as a single disease-specific trigger. Ageing, obesity, metabolic dysfunction, and injury can promote synovial activation, macrophage infiltration, and cytokine production, thereby reducing tissue resilience and enhancing matrix catabolism [43–45, 54]. Impaired inflammatory resolution may also contribute; hyperglycaemia-related CD11b lactylation, for example, has been linked to defective macrophage efferocytosis and sustained synovial inflammation [46].

Lifestyle factors mainly modify risk through body weight, muscle function, mechanical exposure, and systemic metabolism. Physical inactivity can worsen metabolic health, whereas appropriately dosed exercise is generally beneficial. Dietary lipid composition may also matter: palmitate can promote ER stress and inflammatory cartilage responses [16, 55, 56], whereas oleate can attenuate palmitate-induced mitochondrial dysfunction [57]. However, many lifestyle associations remain observational or preclinical and should not be interpreted as deterministic causes.

### Interaction among mechanical, metabolic, and inflammatory risks

The major OA risk factors converge through shared pathways. Mechanical injury may increase matrix permeability and cellular stress; inflammatory mediators can enhance lipid uptake and catabolic signaling; and systemic metabolic disease can provide persistent substrates and signals that further modify local joint metabolism. In experimental systems, FFAs and IL-1β each promote lipid-droplet accumulation, with stronger effects when combined, while high-fat diet and surgically induced OA can synergistically worsen lipid deposition and structural damage [13, 51, 58].

These observations support a multihit model in which intrinsic susceptibility determines the threshold for damage and interacting mechanical, metabolic, and inflammatory stresses determine whether that threshold is crossed. Different combinations of risk factors may therefore converge on similar clinical phenotypes through distinct molecular routes. Major epidemiological patterns and risk factors for OA are summarized in Table 1.

**Table 1:** Major risk factors for osteoarthritis

Risk factor	Main association with OA	Biological relevance	References
Ageing	Strong increase in prevalence with age	Reduced tissue resilience, impaired repair, mitochondrial dysfunction and senescence-associated changes	[1, 2, 34–36]
Sex	Higher overall burden in women, particularly after midlife	Hormonal, anatomical, immune and metabolic differences may modify susceptibility	[1, 20–26]
Genetic susceptibility	Polygenic contribution to OA risk and disease trajectory	Influences skeletal development, matrix homeostasis and tissue repair	[2, 5]
Joint injury / abnormal loading	Major driver of post-traumatic and mechanically mediated OA	Matrix disruption, altered load distribution and persistent tissue stress	[37–40, 59, 60]
Obesity	Strong association, especially with knee OA	Mechanical overload plus adipose-derived metabolic and inflammatory mediators	[2, 3, 11, 12, 41–45]
Diabetes / metabolic syndrome	Associated with increased OA susceptibility	Hyperglycaemia, oxidative/metabolic stress and impaired inflammatory resolution	[9, 46, 47]
Dyslipidaemia	Association reported but inconsistent across cohorts	Local lipid handling may be more informative than serum lipid levels alone	[47–53]
Low-grade inflammation / lifestyle	Can amplify OA susceptibility and progression	Synovial activation, macrophage responses, body weight and mechanical exposure	[38, 43–46, 54–58]

## Molecular pathogenesis of osteoarthritis: a whole-joint perspective

OA is characterized by coordinated pathological remodeling across multiple joint tissues rather than isolated cartilage failure. Cartilage degradation, synovial inflammation, subchondral bone remodeling, altered osteochondral communication, and pathology in menisci and periarticular soft tissues are interconnected through shared mechanical, inflammatory, and metabolic networks [2, 59–64]. Once these processes become mutually reinforcing, initially adaptive responses can evolve into persistent whole-joint dysfunction.

### Articular cartilage degradation and chondrocyte dysfunction

Articular cartilage depends on highly specialized chondrocytes to maintain an extracellular matrix rich in type II collagen and aggrecan. In healthy cartilage, SOX9-centered transcriptional programs preserve chondrocyte identity and support matrix synthesis [65, 66]. OA disrupts this balance by reducing anabolic activity while increasing matrix-degrading enzymes, including matrix metalloproteinases and aggrecanases, leading to progressive proteoglycan loss, collagen-network disruption, and impaired biomechanical function [2, 11].

Chondrocyte dysfunction extends beyond increased matrix catabolism. Single-cell and molecular studies indicate that OA cartilage contains inflammatory, prehypertrophic, hypertrophic, fibrocartilage-like, and other disease-associated chondrocyte states rather than a uniform population of degenerating cells [67–69]. Reactivation of hypertrophic programs involving RUNX2, COL10A1, and MMP13, together with loss of matrix-maintaining regulators such as SOX9, reflects progressive erosion of the stable articular chondrocyte phenotype [66, 70]. These changes reduce reparative capacity and increase responsiveness to inflammatory and mechanical stress.

Matrix disruption alters tissue permeability and the transport of soluble molecules between synovial fluid, cartilage, and subchondral bone [39]. Thus, cartilage damage is not simply an endpoint of OA but contributes actively to the amplification of whole-joint pathology.

### Synovial inflammation and immune dysregulation

Synovial inflammation is an important component of OA and contributes to pain, effusion, and structural progression, although its magnitude varies substantially between patients and disease stages [2, 38]. The OA synovium contains activated fibroblast-like synoviocytes, macrophages, and other immune cells that generate cytokines, chemokines, proteases, and metabolic mediators capable of influencing neighboring cartilage and subchondral tissues [45, 71].

Synovial macrophages are important regulators of both inflammatory activation and resolution. Persistent inflammatory mediators, including IL-1β, TNF-α, and IL-6, can enhance chondrocyte catabolism and matrix degradation, whereas impaired clearance of damaged or apoptotic cells may prolong inflammatory signaling. Recent evidence that altered macrophage metabolism can impair efferocytosis further illustrates the close relationship between metabolic state and inflammatory resolution in OA [46].

Cartilage-synovium communication is bidirectional. Signals released from damaged cartilage can activate synovial cells, whereas synovial mediators can diffuse into cartilage and further modify chondrocyte behavior [71]. This reciprocal circuit helps explain why synovitis can persist after the initiating mechanical insult and why inflammation should be viewed as an integral component of the OA disease network rather than merely a secondary reaction to cartilage loss.

### Subchondral bone remodeling and osteochondral crosstalk

Subchondral bone is another active participant in OA pathogenesis. Altered bone turnover, sclerosis, trabecular remodeling, vascular changes, and osteocyte dysfunction modify both the mechanical and biochemical environment of overlying cartilage [59, 72]. These changes are dynamic and may vary with disease stage, emphasizing that subchondral bone pathology is not simply a late consequence of cartilage degeneration.

The osteochondral interface enables communication between bone and cartilage through structural channels, soluble mediators, and cell-derived signals [73–75]. Osteocytes are particularly well positioned to participate in this process because their dendritic network allows them to sense changes in mechanical and metabolic conditions within subchondral bone and communicate with surrounding cells [76, 77]. Accordingly, alterations in osteocyte function may influence cartilage independently of changes in bone stiffness alone. Bidirectional signaling between subchondral bone and cartilage provides a basis for the metabolic relay mechanisms discussed later in this review.

### Cellular stress, senescence, and regulated cell death

Persistent cellular stress provides a common mechanism linking tissue injury with chronic OA progression. Mitochondrial dysfunction, oxidative stress, endoplasmic-reticulum stress, impaired autophagy, and defective proteostasis can reduce chondrocyte resilience and promote inflammatory and catabolic responses [34, 35, 78]. OA chondrocytes show impaired metabolic flexibility and mitochondrial abnormalities, while disruption of mitochondrial quality-control pathways can compromise mitochondrial integrity and cellular homeostasis [79, 80].

Cellular senescence may add a more persistent component to this stress response. Senescence-associated chondrocyte phenotypes are characterized by impaired reparative capacity and increased production of inflammatory and matrix-degrading mediators [18, 36, 81]. However, stress-associated expression of markers such as p21 does not necessarily establish irreversible senescence. Accordingly, the terms senescence-associated or senescence-like remodeling are preferable when the full biochemical and functional criteria for cellular senescence have not been demonstrated. This distinction is particularly important when considering whether pathological cell states remain therapeutically reversible.

Regulated cell-death pathways further contribute to tissue loss. Apoptosis has long been implicated in OA, while ferroptosis and lipid-peroxidation-associated injury have emerged as additional mechanisms of chondrocyte dysfunction [82]. These pathways interact with mitochondrial, oxidative, and ER stress rather than operating independently. Collectively, chronic cellular stress, senescence-associated remodeling, and regulated cell death reduce the capacity of joint tissues to restore homeostasis and promote persistence of the disease state.

### Major signaling and metabolic networks

The tissue-level abnormalities described above are coordinated by interconnected signaling networks rather than by a single dominant pathway. NF-κB and MAPK pathways integrate inflammatory and stress signals and promote expression of cytokines and matrix-degrading enzymes [83, 84], whereas TGF-β/BMP and WNT-related signaling contributes to chondrocyte differentiation, hypertrophic remodeling, and osteochondral homeostasis [85–87]. Energy-sensing pathways centered on AMPK regulate mitochondrial function, autophagy, and cartilage homeostasis [79, 80, 88, 89], while cytosolic DNA sensing through cGAS-STING links mitochondrial damage to innate immune activation and cartilage degeneration [90–92].

These pathways exhibit extensive crosstalk. Inflammatory signaling can impair mitochondrial function and proteostasis; cellular stress can alter transcriptional and epigenetic programs; and signals originating from synovium or subchondral bone can modify chondrocyte phenotype. Such network-level interactions help explain why OA may continue to progress after the initiating insult has subsided and why targeting a single downstream mediator often fails to fully interrupt disease evolution.

**Fig. 1:**
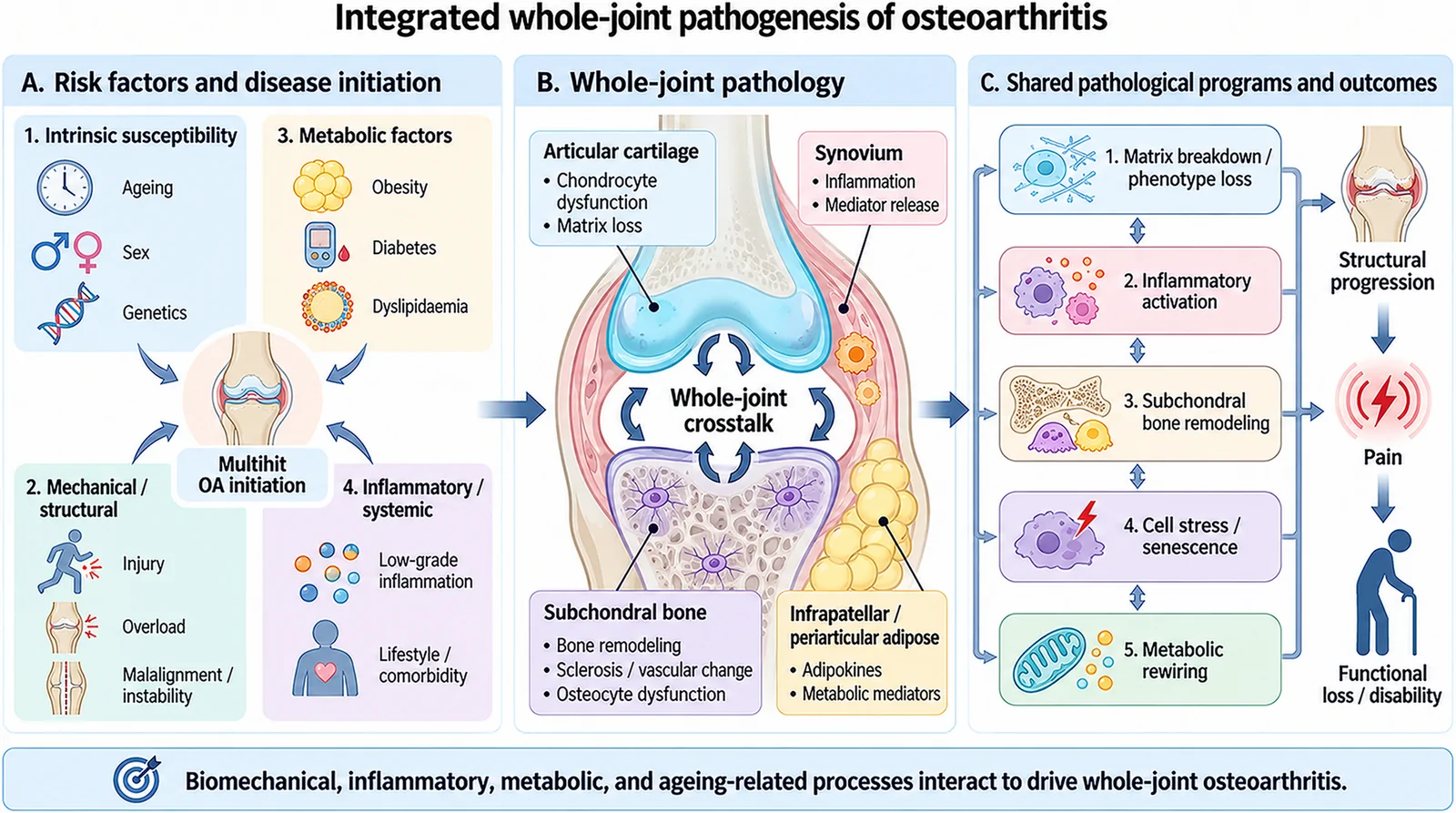
Integrated whole-joint pathogenesis of osteoarthritis. Ageing, genetic susceptibility, mechanical stress or injury, metabolic dysfunction, and inflammatory or systemic factors interact to influence OA initiation and progression. These inputs converge across articular cartilage, synovium, subchondral bone, and periarticular tissues through bidirectional tissue crosstalk. The resulting pathological programs include chondrocyte dysfunction, matrix degradation, synovial inflammation, subchondral bone remodeling, cellular stress, and metabolic reprogramming, ultimately contributing to structural progression, pain, and functional impairment

Metabolic reprogramming is increasingly recognized as an integrative layer within this network. Changes in glycolysis, mitochondrial respiration, amino-acid metabolism, and lipid metabolism can influence energy balance, redox status, metabolite availability, chromatin regulation, and protein stability [34, 93, 94]. Among these processes, lipid metabolism is particularly relevant because lipids can function simultaneously as metabolic substrates, membrane components, signaling mediators, and regulators of cellular state. Taken together, OA can be viewed as a dynamic whole-joint disease in which ageing, biomechanical, metabolic, and inflammatory stresses converge across cartilage, synovium, subchondral bone, and other joint compartments to generate interconnected pathological programs. An integrated overview of these relationships, from disease risk and initiation to tissue pathology and clinical consequences, is presented in Fig. 1.

### Menisci, ligaments, tendons, and osteophyte formation

OA pathology extends beyond cartilage, synovium, and subchondral bone. The meniscus is a load-sharing and shock-absorbing structure, and meniscal tears, extrusion, and altered tissue mechanics are associated with cartilage loss and structural progression. Longitudinal imaging studies support meniscal extrusion as a marker of altered compartmental loading, although the direction and magnitude of this relationship are not uniform across compartments [95–98]. Meniscal pathology therefore belongs in the initiating and propagating phases of knee OA rather than being treated as a secondary radiographic finding. Ligament degeneration and instability similarly alter joint mechanics and are biologically coupled to cartilage, synovial, and subchondral changes [99]. Tendons and entheses contribute through altered force transmission and periarticular inflammation, although their independent causal contribution is less well defined. Osteophytes represent another active tissue response to abnormal mechanical and morphogenetic signaling. TGF-β/BMP-related pathways and altered endochondral ossification programs contribute to osteophyte development, illustrating that new bone formation is an active component of OA remodeling rather than simply a late structural consequence [100–103].

### Pain biology, nociception, and neurovascular remodeling

Pain is a defining clinical outcome of OA, but pain intensity correlates only imperfectly with radiographic structural severity. Multiple joint tissues can contribute nociceptive input, including synovium, periosteum, subchondral bone, ligaments, and inflamed periarticular tissues; articular cartilage itself is normally aneural [104, 105]. Inflammatory mediators, NGF–TrkA signaling, prostaglandins, chemokines, and ion-channel activity sensitize peripheral nociceptors, while persistent nociceptive input contributes to central sensitization and altered pain processing [106–109]. Neurovascular remodeling at the osteochondral junction is particularly relevant because vascular and sensory nerve ingrowth into normally non-innervated regions can couple structural remodeling to pain generation [109–113]. These mechanisms help explain discordance between structural burden and symptoms. Peripheral and central sensitization are not synonymous with advanced structural disease; they can coexist with relatively modest radiographic changes and can influence treatment response. Consequently, pain phenotype should be considered alongside structural and molecular measures when interpreting OA heterogeneity and designing trials [105, 108].

### Mechanical loading and cartilage mechanobiology

Mechanical loading is not simply a background risk factor; it is continuously interpreted by chondrocytes, osteocytes, synovial cells, and periarticular tissues. The biological response depends on magnitude, frequency, duration, loading history, and tissue state. Mechanosensing structures include integrins, primary cilia, cytoskeletal and LINC-complex components, focal-adhesion pathways, and mechanosensitive ion channels such as TRPV4 and PIEZO family channels [64, 114, 115]. Downstream signaling converges on calcium flux, MAPK, NF-κB, Wnt/β-catenin, YAP/TAZ, and metabolic pathways, thereby linking mechanical exposure to matrix turnover, inflammatory activation, and cellular fate. Recent biomechanical work emphasizes the need to combine quantitative loading measurements with longitudinal imaging and clinical outcomes rather than treating mechanics as a static exposure [64, 114–116]. This mechanobiological view also provides a bridge to lipid metabolic coding: mechanical stress can alter lipid-droplet trafficking and cellular energy demand, thereby changing the metabolic context in which lipid signals are interpreted [40].

### Extracellular vesicles and systemic inflammatory context

Extracellular vesicles (EVs) provide an additional route of communication among chondrocytes, synovial cells, macrophages, osteoblast-lineage cells, and other joint compartments. Their cargo includes proteins, nucleic acids, lipids, and metabolites, and EV effects are strongly dependent on cellular origin and disease context [117–119]. Current evidence supports both pathogenic and reparative functions: stressed joint cells can release EVs that reinforce inflammation and matrix catabolism, whereas selected mesenchymal stromal cell-derived EVs can exert immunomodulatory and chondroprotective effects. Synovial-fluid EVs are also being investigated as minimally invasive molecular readouts, but standardization, potency assays, manufacturing, and clinical validation remain unresolved [117–119]. Systemic factors add another layer. The gut–joint axis has been associated with dysbiosis, altered intestinal barrier function, immune activation, and circulating microbial metabolites, but causal relationships in human OA remain incompletely established [120, 121]. These systemic pathways are therefore best regarded as modifiers of the joint environment rather than universal upstream causes.

### Genetics, epigenetics, and disease initiation versus progression

Genetic susceptibility provides a relatively stable component of OA risk, whereas epigenetic and transcriptional states are more dynamic and can integrate ageing, mechanical exposure, inflammation, and metabolism. Recent functional-genomic studies have combined GWAS, chromatin accessibility, single-cell transcriptomics, and spatial profiling to connect risk loci with disease-relevant cell states and regulatory programs [122–125]. Epigenetic mechanisms, including DNA methylation, histone modifications, and non-coding RNAs, can influence chondrocyte differentiation, inflammatory responses, and matrix turnover [121, 126]. These layers should nevertheless be separated conceptually from disease initiation. A molecular change observed in advanced surgical tissue can represent a cause, a consequence, or an adaptive response. Longitudinal biomarker studies after joint injury and before major structural deterioration are therefore particularly valuable for identifying early disease-driving signals [16, 113, 127]. The distinction between initiation and progression is central to therapeutic interpretation because an intervention that modifies an established endpoint does not necessarily reverse the upstream process that initiated disease.

## Lipid metabolic coding in osteoarthritis

The whole-joint mechanisms discussed above are closely linked to metabolic dysregulation. Lipid metabolism, in particular, connects systemic metabolic disturbances with local joint pathology through effects on cellular signaling, organelle homeostasis, and tissue crosstalk. This provides the rationale for examining lipid metabolic coding, focusing on fatty-acid metabolism, lipid synthesis and turnover, cholesterol handling, and their implications for OA progression.

### Concept, terminology, and evidence boundaries

The term lipid metabolic coding is used here as an organizing framework to describe how lipid-derived substrates, metabolites, and lipid-associated stress signals are sensed and processed by joint cells and subsequently translated into molecular and cellular states relevant to OA. It extends beyond conventional lipotoxicity, which primarily denotes cellular injury caused by lipid overload, and is narrower than the broader clinical concept of metabolic OA, which encompasses associations with obesity, diabetes, dyslipidaemia, and other systemic metabolic abnormalities [14, 47]. The framework therefore focuses on the intermediate biological processes that connect lipid exposure with altered cell identity, inflammatory activation, organelle dysfunction, and tissue crosstalk.

The term “coding” is intended to add an organizational level that is not captured fully by lipid metabolic regulation, lipid sensing, or a generic metabolic state. Lipid metabolic regulation describes changes in metabolic pathways; lipid sensing emphasizes receptors and intracellular sensors; lipidomics describes measured lipid composition; lipotoxicity emphasizes injury caused by lipid overload; and metabolic reprogramming describes broader changes in cellular pathway utilization. “Metabolic OA” is a clinical and epidemiological construct that links OA with obesity, diabetes, dyslipidaemia, and related systemic metabolic states. By contrast, lipid metabolic coding focuses on the conditional mapping between lipid inputs and cellular outputs: the same lipid species can produce different responses according to concentration, duration, subcellular routing, cell identity, tissue compartment, disease stage, and concurrent mechanical or inflammatory signals. The framework therefore emphasizes interpretation rather than simply abundance. It also generates experimentally testable predictions—for example, that two joints with similar circulating lipid levels can exhibit different local lipid-processing states, or that altering lipid flux, clearance, or efflux can shift a cellular state without globally suppressing lipid metabolism. The term should thus be understood as a hypothesis-generating framework for organizing these conditional relationships, not as a claim that cells possess a discrete molecular “code” or that the proposed states constitute validated clinical subtypes [125, 127, 128].

Importantly, “coding” is a conceptual metaphor rather than a literal or deterministic biological code. Lipid signals do not generate fixed one-to-one outputs. Their effects depend on lipid species, concentration, exposure duration, cell type, tissue compartment, disease stage, and concurrent mechanical or inflammatory stimuli. The same pathway may be adaptive under transient stress but maladaptive when sustained. Accordingly, lipid metabolic coding should be viewed as context-dependent, probabilistic, and potentially reversible, rather than analogous to digital information processing.

The related concept of PTM memory also requires precise definition. Here, it refers not to the prolonged persistence of a single modified protein, but to a self-reinforcing regulatory state maintained by altered post-translational modification networks, protein turnover, signaling thresholds, and downstream transcriptional programs. Such states may persist beyond the initiating metabolic stimulus but should not be assumed to be irreversible.

Evidence supporting individual components of this framework is heterogeneous. Mechanisms supported by convergent human observations, in vivo perturbation, and functional cellular experiments are considered relatively well established; findings derived predominantly from a single animal model or in vitro system are treated as provisional; and spatial assignments or proposed lipid-associated endotypes without direct metabolic tracing or intervention remain hypothesis-generating. Throughout this section, these distinctions are emphasized explicitly to separate demonstrated mechanisms from mechanistic inference.

### Lipid inputs and lipid-species specificity

Lipid exposure within the OA joint is heterogeneous in both origin and molecular composition. Relevant inputs may derive from circulating lipoproteins and adipose tissue, synovial fluid, local cellular lipid synthesis, membrane turnover, and altered lipid storage or clearance. Synovial fluid is particularly important because it provides a major biochemical interface for avascular cartilage and contains free fatty acids (FFAs), phospholipids, sphingolipids, lipoprotein-associated lipids, and other bioactive lipid species [39, 50, 129]. OA-associated changes in synovial-fluid and cartilage lipid profiles therefore reflect not simply an increase in total lipid abundance, but a remodeling of lipid composition and availability.

A central principle is that not all fatty acids are biologically equivalent. Saturated fatty acids, particularly palmitate, have been linked to pro-inflammatory and stress responses in chondrocytes and can synergize with IL-1β to promote catabolic signaling and endoplasmic-reticulum stress [16, 55, 56]. By contrast, oleate can attenuate palmitate-induced mitochondrial dysfunction in chondrocytes [57], indicating that lipid saturation and molecular structure influence cellular outcome. These observations argue against treating “FFA exposure” as a uniform metabolic signal and support distinguishing individual fatty-acid classes whenever the evidence permits.

Lipidomic studies further implicate phospholipid, sphingolipid, neutral-lipid, and fatty-acid-containing species in OA [50, 130, 131]. However, for many individual sphingolipid or phospholipid species, current evidence remains primarily associative, and their direct causal roles in chondrocyte fate or whole-joint progression are less well established than those of palmitate or cholesterol-related pathways. Lipidomic abundance should therefore not be equated automatically with mechanistic importance.

Cholesterol represents a distinct lipid input because peripheral joint cells cannot degrade it and instead depend on tightly regulated uptake, intracellular trafficking, esterification, and efflux. Its biological consequences may arise not only from intracellular accumulation but also from generation of oxysterol signals and altered nuclear-receptor activity [53, 132]. These cholesterol-specific decoding mechanisms are examined in detail in the cholesterol-handling subsection.

**Fig. 2:**
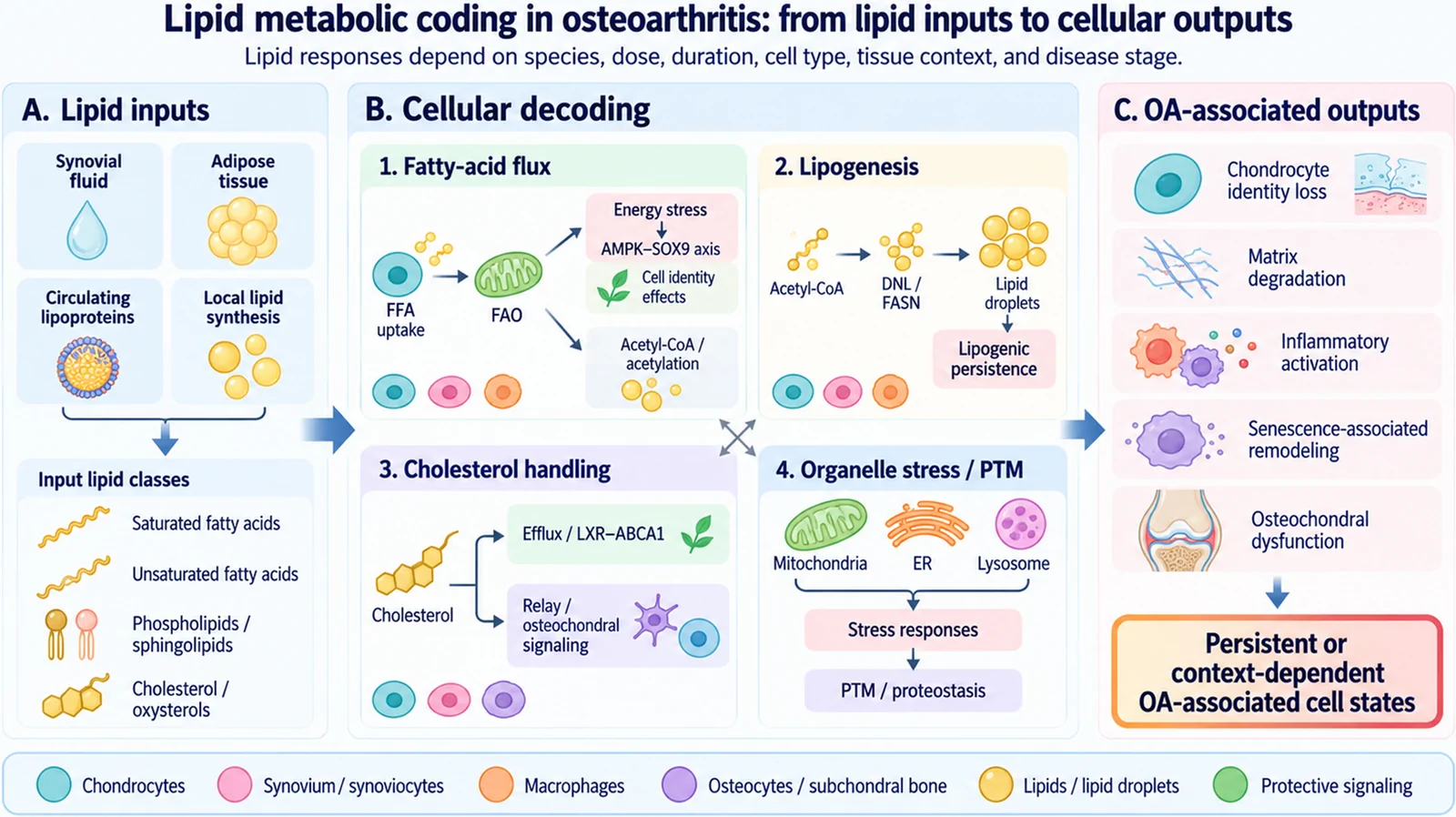
Lipid metabolic coding in osteoarthritis: from lipid inputs to context-dependent cellular outputs. Diverse lipid species derived from systemic and local sources are processed through interconnected cellular decoding modules, including fatty-acid flux, de novo lipogenesis, cholesterol handling, and organelle stress/PTM networks. These processes influence chondrocyte identity, matrix homeostasis, inflammatory activity, senescence-associated remodeling, and osteochondral signaling. The biological consequences of lipid perturbation are shaped by lipid identity, exposure conditions, cellular context, and disease stage, resulting in adaptive, maladaptive, or persistent responses

Thus, the pathogenic relevance of lipid exposure is determined by species identity, source, concentration, cellular handling capacity, and tissue context, rather than by total lipid burden alone. This species-specific view also provides the rationale for Table 2, which summarizes major lipid classes, representative species, joint sources, biological effects, and the strength of supporting evidence. These observations support a context-dependent model in which diverse lipid inputs are interpreted through interconnected metabolic, organelle, and proteostatic modules rather than through fixed one-to-one signaling pathways. An overview of this proposed lipid metabolic coding framework, from lipid inputs through cellular decoding to OA-associated cellular states, is presented in Fig. 2. The major decoding modules are discussed in the following sections.

**Table 2:** Major lipid classes and lipid-related decoding modules in osteoarthritis

Lipid class / input	Main mechanism	OA-related effect	Evidence / references
Saturated fatty acids (e.g., palmitate)	FFA uptake, FAO, ER-stress and inflammatory signaling	Chondrocyte dysfunction and matrix catabolism	Functional preclinical evidence [16, 55, 56]
Unsaturated fatty acids (e.g., oleate, PUFA)	Modulation of fatty-acid stress and mitochondrial responses	Context-dependent protective or modifying effects	Emerging / heterogeneous [57, 58]
Phospholipids / sphingolipids	Membrane remodeling and lipid signaling	Associated with OA-related lipid remodeling	Mainly associative lipidomic evidence [49–51, 130, 131]
Cholesterol / oxysterols	LXR–ABCA1/AIBP and CH25H–CYP7B1–RORα pathways	Altered cholesterol homeostasis and catabolic signaling	Mechanistic evidence [19, 53, 132, 133]
Acetyl-CoA / DNL	FAO–AMPK–SOX9 and ACOT12–FASN regulation	Loss of chondrocyte identity and lipogenic persistence	Emerging mechanistic evidence [13, 52, 134–143]
Lipid droplets	PLIN3–HSC70–LAMP2A and lysosomal clearance	Persistent lipid accumulation and senescence-associated remodeling	Predominantly preclinical [40, 51, 144, 145]
Lipid-sensitive PTM networks	Phosphorylation, ubiquitination, SUMOylation and palmitoylation	Altered protein stability and persistent cellular signaling	Emerging mechanistic evidence [13, 18, 46, 81, 134, 146–148]

### Fatty acid flux, Acetyl-CoA, and loss of chondrocyte identity

Fatty-acid metabolism provides one of the clearest examples of how a lipid input can be translated into a cell-state change in osteoarthritis. Chondrocytes can actively increase fatty-acid uptake under lipid-rich conditions, accompanied by induction of lipid-handling genes such as CD36, FATP5, FABP3, and LIPE [13, 149]. In experimental OA, this enhanced uptake is coupled to increased fatty-acid oxidation (FAO) and accumulation of fatty acid-derived acetyl-CoA, indicating that excess lipid is not merely stored but is redirected into metabolic and regulatory pathways [13].

A central consequence of this shift is disruption of the AMPK-SOX9 axis. Excessive FAO increases cellular energy production and suppresses AMPK activity, thereby weakening AMPK-dependent stabilization of SOX9 [13]. This is mechanistically important because SOX9 is a core determinant of the differentiated articular chondrocyte phenotype and is required for sustained expression of matrix genes including COL2A1 and ACAN [65, 66]. Under FFA exposure, SOX9 protein abundance decreases more prominently than SOX9 mRNA, suggesting a post-translational mechanism. FFA treatment increases SOX9 ubiquitination, whereas AMPK activation promotes SOX9 phosphorylation and protein stability, supporting a model in which reduced AMPK activity facilitates ubiquitin-dependent SOX9 degradation [134]. Thus, FAO can function not only as a fuel-utilization pathway but also as a metabolic switch linking lipid excess to erosion of chondrocyte identity.

Acetyl-CoA provides a second regulatory layer. Increased FAO is accompanied by broader protein acetylation and acetylome remodeling, including acetylation of the mitochondrial FAO enzyme HADHA, which may further reinforce oxidative lipid flux [13, 135]. Acetyl-CoA can also contribute to histone acetylation and transcriptional regulation [136–138]. However, this relationship is likely to depend on subcellular compartmentalization, because mitochondrial acetyl-CoA and the cytosolic/nuclear pools available for chromatin modification are metabolically connected but not interchangeable. In OA, altered H3K27ac patterns have been observed at cartilage anabolic and catabolic regulatory regions, but locus-specific causal links between lipid-derived acetyl-CoA and individual OA genes remain incompletely defined [139–142].

The evidence therefore supports a model in which fatty-acid flux can influence both protein stability and epigenetic regulation, but the strength of evidence differs across levels. The FAO-AMPK-SOX9 relationship is supported by functional perturbation in experimental models, whereas direct demonstration of the complete pathway in human OA cartilage, particularly the metabolic origin and genomic destination of acetyl-CoA-derived acetylation, remains limited. These distinctions are important when interpreting fatty-acid flux as a mechanistic component of lipid metabolic coding rather than as a universally established upstream driver of OA.

### De novo lipogenesis, lipid droplets, and senescence-associated remodeling

In addition to importing extracellular fatty acids, chondrocytes can generate lipid signals through de novo lipogenesis (DNL). Cytosolic acetyl-CoA is converted through ACACA and FASN into fatty acids that can subsequently be incorporated into membrane lipids, neutral lipids, or lipid droplets [51, 143]. Under physiological conditions, this pathway supports membrane and metabolic homeostasis, whereas excessive DNL may contribute to pathological lipid accumulation. The PPARα-ACOT12 axis provides an important regulatory checkpoint: ACOT12 hydrolyses cytosolic acetyl-CoA and thereby limits substrate availability for lipogenesis, while loss of ACOT12 in OA cartilage increases acetyl-CoA, ACACA, FASN, and SCD1 and promotes lipid accumulation and matrix-degrading responses. Restoration of ACOT12 alleviates these abnormalities in human OA chondrocytes and experimental OA, supporting a functional role for this pathway [52].

Persistence of the lipogenic program may depend on regulation of FASN protein stability. CircRREB1 is increased in senescent chondrocytes and promotes FASN accumulation, lipid deposition, p21 expression, and MMP13-associated matrix catabolism [18]. Mechanistically, CircRREB1 reduces acetylation-dependent ubiquitination of FASN while enhancing RanBP2-mediated SUMOylation, thereby stabilizing the enzyme and sustaining lipogenic activity [18, 146–148]. These findings provide a plausible basis for lipogenic persistence, but the resulting phenotype should be described cautiously. Increased p21, lipid accumulation, and catabolic signaling support a senescence-associated or senescence-like state, but do not by themselves establish irreversible cellular senescence. Accordingly, “lipogenic memory” is best interpreted as a potentially self-reinforcing metabolic and proteostatic state rather than permanent cell-state fixation.

Lipid droplets (LDs) represent the storage arm of this process. LD formation may initially protect cells by sequestering excess fatty acids, but persistent accumulation can prolong intracellular lipid exposure when trafficking and clearance are impaired [57, 131, 144]. In OA models, abnormal mechanical stress disrupts microtubule-dependent LD transport and the PLIN3-HSC70-LAMP2A axis, leading to defective LD turnover and cartilage degeneration; low-dose docetaxel partially restores this process [40]. Importantly, this mechanism should be distinguished from canonical lipophagy. Macroautophagic lipophagy refers to lysosomal degradation of LD material, whereas chaperone-mediated autophagy (CMA) can remove LD-associated proteins such as PLIN family members and thereby facilitate subsequent lipid mobilization [144, 145]. This distinction avoids treating all lysosome-dependent LD turnover as a single process.

Together, DNL, FASN stabilization, and defective LD clearance provide complementary mechanisms by which transient metabolic stress may be prolonged within chondrocytes. However, whether LD-mitochondrial or LD-ER contact sites directly contribute to OA-specific lipid coding remains insufficiently established in the evidence base used here and requires further mechanistic validation.

### Cholesterol handling and osteochondral metabolic relay

Cholesterol represents a distinct lipid-processing challenge in OA because peripheral joint cells cannot degrade it and therefore depend on tightly regulated uptake, intracellular trafficking, esterification, and efflux. In chondrocytes, the balance between protective cholesterol-export programs and maladaptive cholesterol-sensitive signaling appears to determine whether cholesterol stress is buffered or translated into catabolic responses. The AIBP-ABCA1/ApoA1-LXR axis promotes cholesterol efflux and limits intracellular accumulation, whereas reduced AIBP expression and impaired LXR signaling have been associated with the catabolic phenotype of OA cartilage [53, 133]. Conversely, the CH25H-CYP7B1-RORα axis converts cholesterol imbalance into oxysterol-dependent transcriptional signaling that promotes matrix-degrading programs and suppresses cartilage homeostasis [132]. These pathways are therefore better viewed as opposing components of a cholesterol-handling network rather than as isolated mechanisms.

Cholesterol stress may also be propagated across the osteochondral unit rather than acting solely within chondrocytes. In experimental OA, cholesterol accumulation is associated with activation of inflammatory pathways including cGAS-STING, yet isolated chondrocytes do not fully reproduce this response after cholesterol exposure alone [19]. This tissue-context dependence led to the identification of subchondral osteocytes as potential intermediaries. Cholesterol-loaded osteocytes exhibit mitochondrial dysfunction and can transfer mitochondria to chondrocytes through intercellular nanotubular structures, increasing cytosolic mitochondrial DNA and activating cGAS-STING-dependent inflammatory and matrix-degrading responses [19]. Inhibition of mitochondrial trafficking or STING signaling attenuates these effects in experimental systems, supporting a functional role for the relay mechanism.

However, the evidence boundary is important. The osteocyte-to-chondrocyte mitochondrial relay is supported predominantly by recent mechanistic and mouse in vivo studies, whereas its prevalence, magnitude, and clinical relevance in human OA remain uncertain. It should therefore be presented as an emerging osteochondral mechanism rather than an established universal pathway.

Several therapeutic entry points arise from this model. Enhancing cholesterol efflux through AIBP/LXR-ABCA1 signaling may reduce intracellular cholesterol burden, whereas modulation of the NUDT8-CoA-pantethine axis may preserve osteocyte mitochondrial function under cholesterol stress [19, 53]. Pantethine improved mitochondrial respiration and attenuated OA pathology in experimental models, but its translational potential remains limited by the systemic importance of CoA metabolism and the need for joint- or cell-specific delivery [19]. These strategies are therefore best considered mechanistically informed candidates for future precision intervention rather than clinically established disease-modifying therapies.

### Organelle stress and lipid-sensitive PTM networks

Lipid stress is processed not only through individual metabolic pathways but also through the collective buffering capacity of mitochondria, the endoplasmic reticulum (ER), lipid droplets, and lysosomes. A useful integrative model is that joint cells possess an organelle stress threshold: below this threshold, excess lipids can be oxidized, stored, trafficked, or cleared as part of an adaptive response; once buffering capacity is exceeded, mitochondrial dysfunction, ER stress, impaired lysosomal clearance, and oxidative injury can amplify inflammatory and catabolic signaling [34, 82, 144, 145, 150]. This model emphasizes that lipid accumulation is not intrinsically pathological; its consequences depend on whether organelle homeostasis can be restored.

Post-translational modification (PTM) networks provide a second layer through which metabolic stress can influence the duration and reversibility of cellular responses. Rather than acting independently, PTMs often determine protein stability, localization, and signaling competence through crosstalk. In chondrocytes, the SOX9 axis illustrates phosphorylation-ubiquitination competition: reduced AMPK-dependent phosphorylation facilitates SOX9 ubiquitination and destabilization during excessive fatty-acid oxidation [13, 134]. The FASN axis provides a complementary example, in which reduced acetylation-dependent ubiquitination together with enhanced SUMOylation promotes FASN stabilization and persistent lipogenic activity [18, 147, 148]. These mechanisms suggest that metabolic inputs can be translated into altered protein turnover rather than merely transient changes in enzyme activity.

PTM-dependent regulation also extends beyond chondrocytes. In synovial macrophages, hyperglycaemia-associated CD11b lactylation impairs efferocytosis and may prolong inflammatory activity [46], whereas reduced ZDHHC11-mediated palmitoylation of APOD facilitates APOD ubiquitination and degradation, promoting senescence-associated signaling in chondrocytes [81]. Importantly, not all of these PTMs are directly generated by lipid metabolites; rather, they form part of a broader metabolically responsive proteostatic network that intersects with lipid stress.

Accordingly, PTM memory should be interpreted as persistence of a self-reinforcing regulatory network involving altered protein modification and turnover, not as permanent retention of a single modified protein. Whether such states are reversible, stage-dependent, or clinically targetable remains unresolved and will require integrated metabolic, proteomic, and spatial validation.

### Spatial remodeling and candidate lipid-associated OA endotypes

The biological effects of lipid stress are unlikely to be spatially uniform across the osteoarthritic joint. Articular cartilage zones differ in matrix composition, oxygen tension, mechanical exposure, and proximity to synovial fluid or subchondral bone, creating distinct metabolic microenvironments [151]. Superficial chondrocytes may be more directly exposed to synovial lipid species [152], whereas deeper cartilage may be more influenced by matrix permeability and osteochondral signals [153]. Single-cell and spatial transcriptomic studies have identified inflammatory, prehypertrophic, hypertrophic, and fibrocartilage-like chondrocyte populations in OA [67, 69, 154], but these datasets generally do not directly measure lipid abundance, metabolic flux, or PTM states in the same spatial context. Lipid-associated spatial remodeling should therefore remain a hypothesis-generating concept pending direct multi-omic validation.

Within this framework, four candidate lipid-associated OA endotypes can be considered: a fatty-acid-flux-dominant state characterized by increased lipid uptake and FAO-related identity loss; a lipogenic-senescence-associated state involving DNL and FASN persistence; a lipid-droplet-clearance-defective state reflecting impaired intracellular lipid turnover; and a cholesterol-handling/relay state involving defective efflux and osteochondral signaling. These categories are mechanistic working models rather than validated clinical subtypes.

Importantly, these endotypes are unlikely to be mutually exclusive or temporally fixed. Early OA may be dominated by lipid exposure and compensatory metabolic adaptation, whereas intermediate or advanced disease may show increasing lipid-droplet persistence, senescence-associated remodeling, or osteochondral inflammatory relay. Obesity, diabetes, and dyslipidaemia may further generate mixed profiles within the same patient, and distinct spatial niches within a single joint may simultaneously exhibit different dominant lipid-processing programs.

**Fig. 3:**
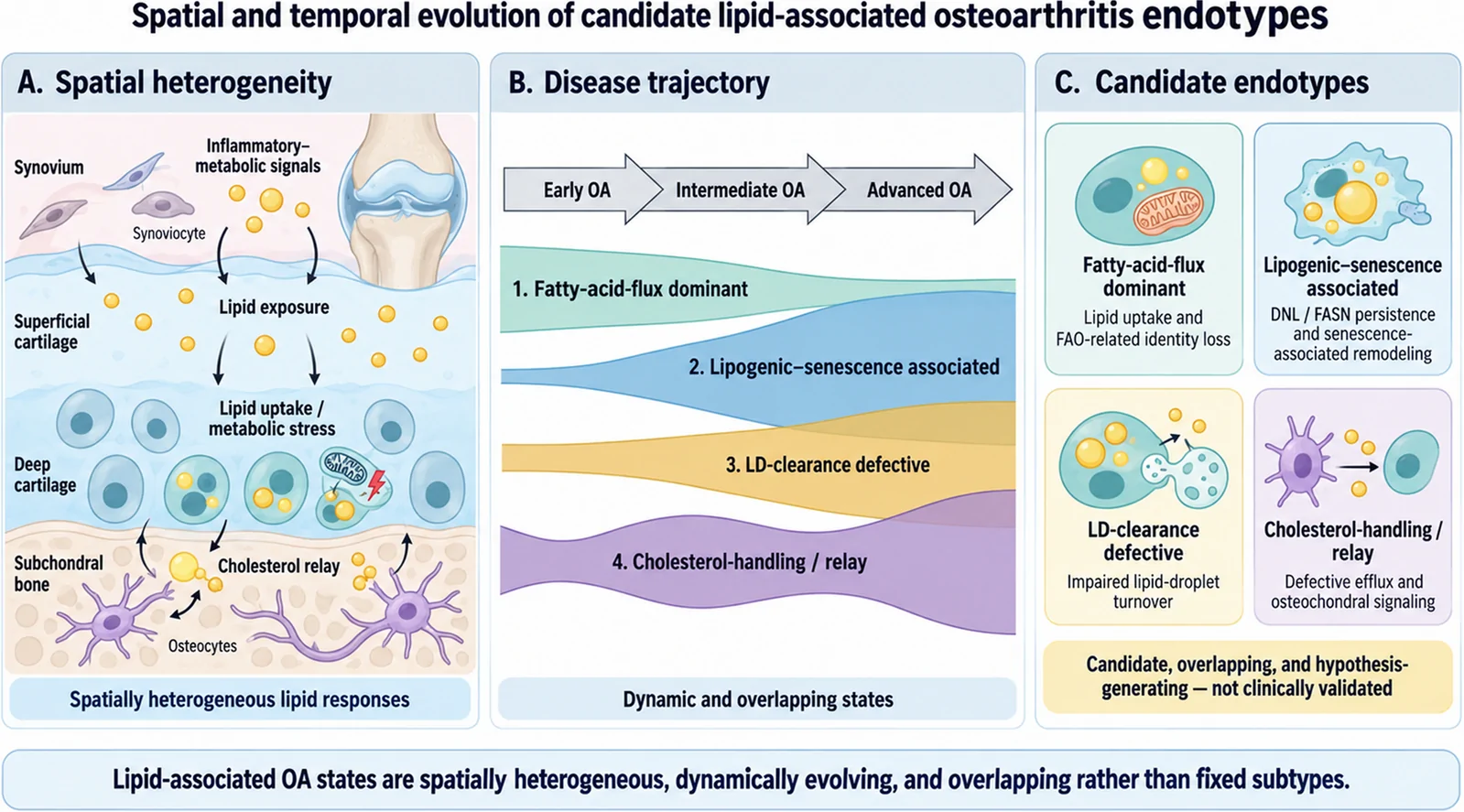
Spatial and temporal evolution of candidate lipid-associated osteoarthritis endotypes. Lipid-associated responses may vary across synovium, superficial and deep cartilage, and subchondral bone and may change dynamically during OA progression. Four candidate endotypes—fatty-acid-flux-dominant, lipogenic–senescence-associated, LD-clearance-defective, and cholesterol-handling/relay—are proposed as overlapping and potentially stage-dependent biological states. Their spatial distribution and relative predominance may be influenced by systemic metabolic status, mechanical stress, inflammation, and other contextual factors. These endotypes remain hypothesis-generating and are not clinically validated classifications

Future validation will require a prioritized biomarker architecture rather than long lists of heterogeneous markers. Clinically accessible measures such as synovial-fluid lipid profiles could form a first layer; tissue-level proteins and pathway readouts a second; and spatial lipidomics, PTM profiling, and metabolic tracing a research-level third layer. These candidate endotypes should only be considered clinically meaningful if they prove reproducible, dynamic, and predictive of disease trajectory or therapeutic response. Collectively, these observations suggest that lipid-associated OA states are spatially heterogeneous and dynamically evolve across disease stages rather than forming fixed, mutually exclusive subtypes. Candidate lipid-associated OA endotypes and their proposed biomarkers are summarized in Table 3. A conceptual overview of their proposed spatial distribution and temporal evolution is presented in Fig. 3.

**Table 3:** Candidate lipid-associated osteoarthritis endotypes

Candidate endotype	Core mechanism	Candidate biomarker domains	Potential intervention	References
Fatty-acid-flux-dominant	Increased lipid uptake and FAO with impaired AMPK–SOX9 homeostasis	Clinically accessible: synovial FFA profile; tissue/pathway: CD36/FABP, SOX9	Restore metabolic balance / AMPK–SOX9 signaling	[13, 49, 50, 134, 149]
Lipogenic–senescence-associated	Increased DNL and FASN persistence	Tissue/pathway: FASN, ACACA, CircRREB1, p21; research: lipid-droplet burden	Modulate DNL/FASN and proteostatic persistence	[18, 51, 52, 146–148]
LD-clearance-defective	Impaired lipid-droplet trafficking and lysosomal/CMA-dependent clearance	Research/tissue: LD burden, PLIN3, HSC70, LAMP2A	Restore lipid-droplet trafficking and clearance	[40, 57, 131, 144, 145]
Cholesterol-handling / relay	Defective cholesterol efflux and osteochondral inflammatory relay	Tissue/pathway: ABCA1/AIBP, LXR/RORα; research: cholesterol-related readouts	Enhance cholesterol efflux or interrupt osteochondral relay	[19, 48, 53, 132, 133]
Evidence boundary	Proposed, overlapping and stage-dependent mechanistic states	Markers are research candidates rather than validated diagnostic biomarkers	No endotype-directed therapy is clinically established

### Evidence boundaries and competing interpretations

Several boundaries should constrain interpretation of the lipid metabolic coding framework. First, lipid remodeling in OA should not be assumed to be uniformly causal. Much of the human evidence is cross-sectional and derived from surgically obtained, advanced OA tissues, making it difficult to distinguish disease-initiating signals from adaptive responses or secondary consequences of matrix failure [3, 49, 50, 130, 155]. Mechanistic studies in rodents and cultured cells provide stronger causal inference but cannot fully reproduce the chronic, ageing-associated, and heterogeneous nature of human OA [156]. Accordingly, individual pathways should be interpreted according to the level and biological context of supporting evidence.

Second, systemic lipid status and local joint lipid biology are not interchangeable. Circulating cholesterol and lipoprotein concentrations do not consistently predict structural progression or symptoms [48, 157], and systemic lipid-lowering approaches have shown inconsistent associations with OA outcomes. These discrepancies may reflect differences in local lipid exposure, cartilage permeability, tissue-specific uptake and efflux, disease stage, and metabolic background. Thus, the framework does not imply that hyperlipidaemia alone defines lipid-associated OA.

Third, the “coding” metaphor has inherent limitations. Biological lipid responses are neither digital nor deterministic; they are dynamic, stochastic, context-dependent, and potentially reversible. Similarly, the candidate lipid-associated endotypes proposed here should not be regarded as alternatives to inflammatory, biomechanical, fibrotic, or senescence-related OA frameworks. Rather, lipid-processing abnormalities may overlap with and modify these broader biological programs within the same patient or joint.

Finally, current technologies remain insufficient to directly resolve many proposed spatial mechanisms. Spatial transcriptomics does not measure lipid abundance or metabolic flux, conventional lipidomics provides limited cellular localization, and isotope tracing is difficult to implement in human cartilage. Validation will therefore require longitudinal human studies integrating spatial lipidomics, metabolic tracing, transcriptomics, proteomics, PTM profiling, and functional perturbation. Until such evidence is available, lipid metabolic coding should be considered a testable mechanistic framework rather than an established clinical classification.

### Biomarker hierarchy and evidence grading

Candidate markers should be separated according to what they actually measure. Proximal, clinically accessible signals, such as synovial-fluid lipid species or circulating proteins, can support patient-level stratification but are influenced by systemic comorbidities and sampling conditions. Tissue-level markers, including CD36/FABP, SOX9, FASN, ACACA, PLIN3, HSC70/LAMP2A, and ABCA1/AIBP, are more mechanistically informative but generally require tissue acquisition or specialized assays. Spatial lipidomics, metabolic tracing, PTM profiling, and direct measurement of lipid-droplet flux remain primarily research tools [125, 127, 158, 159]. We therefore distinguish three evidence classes: (i) clinically accessible candidate biomarkers, (ii) tissue/pathway markers that support mechanistic endotyping, and (iii) research-only markers requiring prospective validation. A marker should not be considered a clinically useful biomarker solely because it changes in OA tissue; clinical utility requires analytical reproducibility, association with a defined outcome, incremental predictive value beyond established clinical variables, and prospective validation [125, 127, 128, 159].

## Therapeutic progress in osteoarthritis

Current OA management primarily aims to reduce pain and maintain function, whereas effective treatments that reliably modify structural disease progression remain limited [2, 11, 38]. The biological heterogeneity described above further complicates therapeutic development, because interventions targeting a single pathway may benefit only selected disease stages or mechanistic subgroups. Therapeutic progress should therefore be viewed as a continuum from symptom control and biomechanical management to emerging disease-modifying and mechanism-directed strategies.

### Non-pharmacological interventions

Non-pharmacological management remains the foundation of OA care. Patient education, structured exercise, muscle strengthening, physical activity, and weight management can improve pain, mobility, and function [160, 161]. In patients with overweight or obesity, weight reduction may additionally decrease mechanical loading and improve systemic metabolic health. Biomechanical interventions, including selected bracing, assistive devices, and rehabilitation programs, may benefit patients with specific alignment or functional abnormalities. Importantly, exercise should be regarded as a therapeutic physiological stimulus rather than avoided because of concerns about cartilage “wear”.

### Current pharmacological therapies

Pharmacological treatment remains predominantly symptom-directed. Topical or oral non-steroidal anti-inflammatory drugs are commonly used for pain control, while other analgesic approaches are selected according to symptoms, comorbidities, and safety considerations. These therapies can improve pain and function but do not reliably prevent structural progression.

### Intra-articular and interventional approaches

Intra-articular corticosteroids can provide temporary relief in selected patients, whereas evidence for hyaluronic acid, platelet-rich plasma, and other injectable interventions varies according to formulation, patient selection, and outcome assessment [11]. Such heterogeneity may partly reflect underlying biological differences among patients rather than technical variation alone. Future interventional strategies may therefore benefit from biomarker- or phenotype-guided selection rather than uniform application across radiographically defined OA populations.

### Surgical management

Surgery remains an essential option when advanced structural damage produces persistent pain and functional limitation despite conservative treatment. Joint-preserving procedures may be considered in selected patients with correctable biomechanical abnormalities [162, 163], whereas arthroplasty provides substantial symptomatic and functional benefit in advanced knee or hip OA [164, 165]. However, surgery addresses the consequences of established disease rather than the molecular processes that initiate progression, emphasizing the need for effective interventions at earlier stages.

### Emerging disease-modifying and regenerative therapies

Disease-modifying OA drug (DMOAD) development aims to preserve or restore joint structure while producing clinically meaningful symptomatic benefit. Anabolic approaches targeting cartilage repair are particularly attractive. Sprifermin, a recombinant FGF18 analogue, has demonstrated increases in cartilage thickness in clinical studies [166, 167], although structural improvement has not translated consistently into symptomatic benefit, illustrating the challenge of linking imaging modification to outcomes that matter to patients. More broadly, recent reviews conclude that no intra-articular regenerative DMOAD has yet established routine clinical efficacy [168].

Other strategies target signaling pathways involved in cartilage remodeling. Lorecivivint, an intra-articular CLK/DYRK inhibitor that modulates Wnt-related signaling, progressed to phase III evaluation [169, 170]. However, the OA-10 phase III trial did not meet its primary pain endpoint in the overall population, although exploratory analyses suggested possible benefit in patients with less severe structural disease [169]. More recent synthesis of six randomized trials likewise indicates modest or inconsistent symptomatic effects without convincing structural improvement [171]. These findings underscore an important translational lesson: therapeutic efficacy may depend on disease stage and patient selection rather than target validity alone.

Regenerative approaches involving growth factors, cell-based therapies, biomaterials, and gene or RNA delivery are also advancing, with encouraging preclinical and early clinical signals but unresolved issues regarding durability, delivery, safety, and reproducibility [172]. Future DMOAD trials will therefore need to align molecular targets, disease stage, structural endpoints, and clinically meaningful outcomes more precisely.

### Metabolic, anti-obesity, and anti-diabetes interventions

Metabolism-directed treatment is particularly relevant to patients with obesity, insulin resistance, or diabetes. Metformin has shown chondroprotective effects in experimental OA through AMPK-related metabolic regulation and has already been incorporated into the mechanistic literature [173]. Recent clinical meta-analyses also suggest reductions in knee pain and possibly joint-replacement risk, particularly in metabolically complex or overweight populations, although study heterogeneity and the limited number of high-quality randomized trials preclude defining metformin as an established DMOAD [174].

Anti-obesity therapies provide another emerging strategy. In the STEP 9 randomized trial, once-weekly semaglutide in patients with obesity and symptomatic knee OA produced substantially greater weight loss and improvement in knee pain than placebo over 68 weeks [175]. Whether this benefit primarily reflects weight reduction, systemic anti-inflammatory effects, or direct actions within joint tissues remains unresolved, and structural disease modification has not been established. This distinction will be important when defining metabolic OA phenotypes.

The distinction between direct joint actions and indirect systemic effects is important when interpreting metabolic interventions. Metformin has experimental evidence for direct chondroprotective actions through AMPK-related pathways, but human evidence does not establish that its clinical effects arise from direct drug exposure within cartilage [173, 176]. GLP-1 receptor agonists such as semaglutide have clinical evidence for weight loss and pain improvement in people with obesity and knee OA, but the contribution of direct joint signaling remains unresolved [175, 177]. Weight loss itself reduces mechanical loading and can improve systemic metabolic and inflammatory status, making it difficult to attribute structural or symptomatic changes to a single molecular pathway [177–179]. Exercise acts predominantly through neuromuscular, biomechanical, and systemic metabolic mechanisms rather than selective pharmacological modulation of a joint pathway [179, 180]. Bariatric interventions similarly produce large systemic and mechanical changes, and any joint benefit should therefore be interpreted as an integrated consequence of weight reduction and metabolic improvement rather than evidence for a specific intra-articular lipid mechanism.

### Why mechanistically plausible systemic interventions can fail in OA

Negative or inconsistent clinical results do not necessarily invalidate a molecular mechanism. Clinical failure may reflect inappropriate patient selection, advanced disease stage, insufficient intra-articular target engagement, compensatory signaling, or insensitive endpoints. Adaptive lipid remodeling, inadequate treatment duration, and selection of the wrong metabolic target may also contribute. These possibilities provide a useful interpretation framework for inconsistent associations between circulating lipid measures, lipid-lowering interventions, and OA outcomes. They also reinforce the distinction between target validity and drug efficacy: a pathway can be causal in a defined tissue or stage without being effectively modified by a systemic intervention in an unselected OA population [120, 177, 181–183]. Future trials should therefore document target engagement, stratify by disease stage and metabolic phenotype, and predefine structural as well as symptomatic endpoints.

### Lipid-directed precision therapeutics

The lipid metabolic coding framework suggests that treatment should aim to reset maladaptive lipid-processing programs rather than globally suppress lipid metabolism. Potential interventions can be mapped to the dominant dysfunctional module. Excessive fatty-acid flux may be addressed through restoration of AMPK-SOX9 homeostasis [13, 173, 184], whereas pathological DNL may be targeted through the PPARα-ACOT12-FASN axis [52]. Impaired lipid-droplet clearance represents a distinct opportunity: experimental restoration of microtubule-dependent PLIN3-HSC70-LAMP2A function reduced lipid accumulation and cartilage degeneration [40].

Cholesterol-related strategies include enhancement of AIBP/LXR-ABCA1-mediated efflux [53], modulation of the CH25H-CYP7B1-RORα pathway [132, 185], and preservation of osteocyte mitochondrial competence through the NUDT8-CoA-pantethine axis [19]. PTM and proteostasis pathways, including ZDHHC11-dependent APOD palmitoylation, provide additional experimental targets [81]. However, most of these approaches remain at the mechanistic or preclinical stage, and their systemic metabolic importance raises concerns regarding off-target effects.

**Fig. 4:**
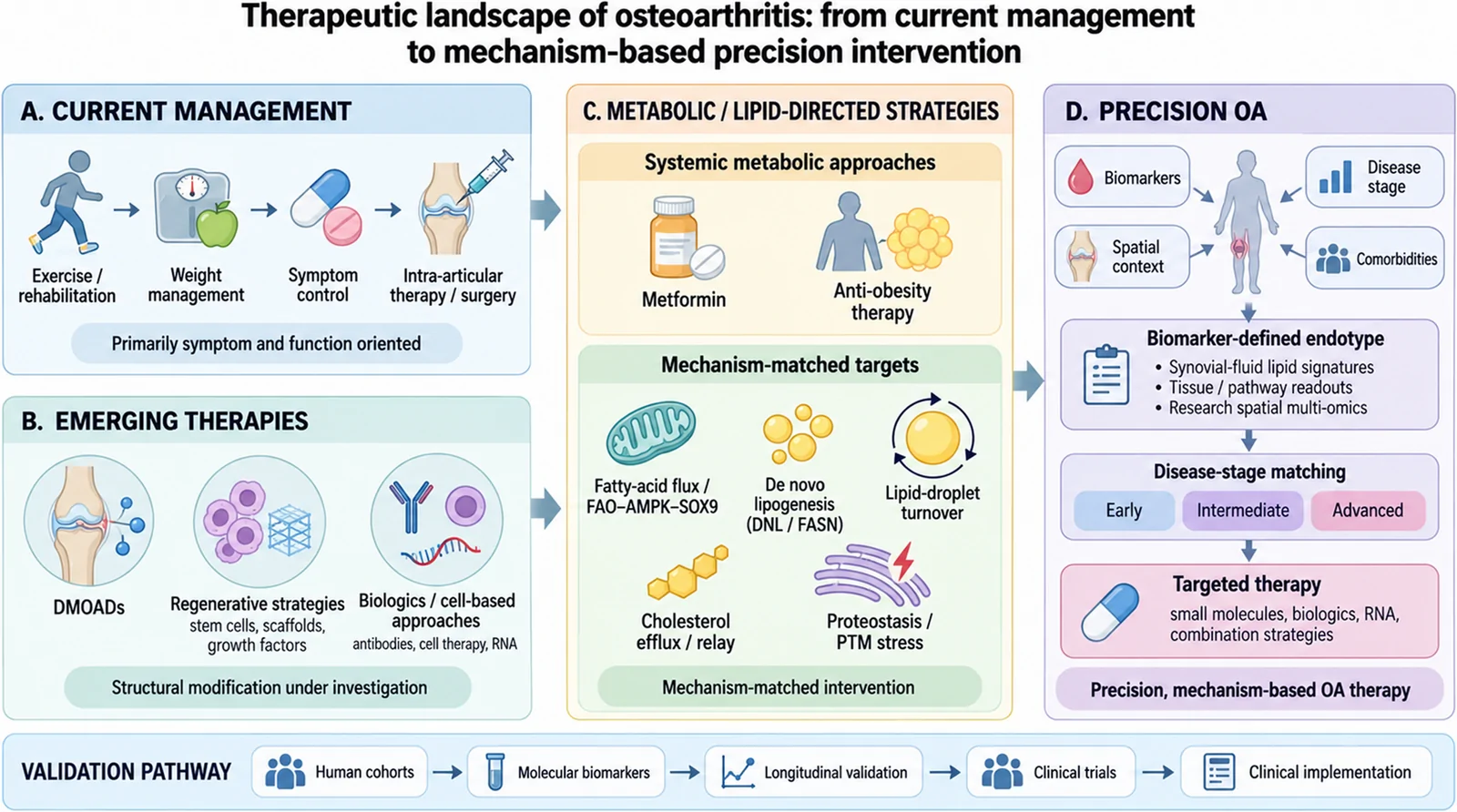
Therapeutic landscape of osteoarthritis: from current management to mechanism-based precision intervention. Current OA management primarily targets symptoms, function, and mechanical burden, whereas emerging approaches aim to modify structural disease through DMOADs, regenerative strategies, and metabolic interventions. Lipid-directed approaches target specific abnormalities in fatty-acid flux, de novo lipogenesis, lipid-droplet turnover, cholesterol handling, or PTM/proteostasis. Future precision therapy will require biomarker-defined patient selection, disease-stage matching, and validation through longitudinal human studies and clinical trials

For this reason, local delivery may be particularly important. Cartilage-penetrating carriers, intra-articular formulations, and cell- or tissue-selective metabolic modulators could increase therapeutic exposure within the joint while minimizing disruption of physiological lipid metabolism elsewhere [186]. Ultimately, lipid-directed treatment will require pairing a dominant lipid-processing abnormality with an appropriate intervention and disease stage, converting the concept of metabolic targeting from broad lipid lowering to mechanism-based precision therapy. Current and emerging OA therapies and their translational evidence are summarized in Table 4. The evolving therapeutic landscape, together with the validation steps required for clinical translation, is summarized in Fig. 4.

**Table 4:** Current and emerging therapies for osteoarthritis

Therapeutic strategy	Representative intervention	Evidence stage	Main effect / key limitation	References
Non-pharmacological	Exercise, rehabilitation, weight management	Clinical standard	Improves pain and function; structural modification remains uncertain	[11, 161, 176, 179, 187, 188]
Symptomatic pharmacotherapy	NSAIDs and other analgesic strategies	Clinical standard	Symptom relief; does not reliably prevent structural progression	[11, 38, 187, 188]
Intra-articular / surgical	Corticosteroids, selected injections, joint-preserving surgery, arthroplasty	Clinical standard / variable	Useful for selected patients; largely addresses symptoms or established structural disease	[11, 162–165, 187, 188]
Candidate DMOAD / anabolic	Sprifermin	Clinical trials	Increases cartilage thickness; symptomatic benefit inconsistent	[166, 167]
Candidate DMOAD / signaling	Lorecivivint	Phase III	Overall benefit inconsistent; patient selection and disease stage may matter	[169–171]
Regenerative / biologic	MSC-, exosome-, PRP-, gene/RNA-based approaches	Preclinical to early clinical	Promising repair signals; durability, delivery and reproducibility remain unresolved	[117–119, 172]
Metabolic	Metformin	Preclinical + clinical evidence	Potential pain and disease-related benefit; not an established DMOAD	[173, 174]
Anti-obesity / metabolic	Semaglutide	Randomized clinical trial	Preclinical + clinical evidence; direct joint versus indirect systemic effects remain unresolved	[175]
Lipid-directed precision strategies	FAO/AMPK–SOX9, DNL/FASN, LD clearance, cholesterol efflux/relay, PTM pathways	Mainly preclinical	Mechanistically attractive; human target engagement, biomarker selection, and delivery remain to be validated	[13, 19, 40, 52, 53, 81, 132, 173, 184–186, 189]

## Future perspectives

The major challenge in OA research is no longer simply to identify additional disease-associated pathways, but to determine which mechanisms are active in particular patients, joint compartments, and disease stages. Future progress will therefore depend on integrating clinical phenotypes with molecular endotypes, spatial and longitudinal disease mapping, and mechanism-guided intervention.

### From clinical phenotypes to molecular endotypes

Current OA classification remains dominated by anatomical site, symptoms, and radiographic severity, none of which fully captures the biological processes driving disease progression or therapeutic response. Molecular endotyping offers a potential route toward more precise stratification, but proposed inflammatory, metabolic, biomechanical, senescence-associated, and lipid-processing endotypes should not be treated as fixed or mutually exclusive categories. The same patient may harbor several dominant programs simultaneously, and their relative importance may change over time.

The next step is therefore to identify reproducible molecular signatures that predict clinically meaningful outcomes. Candidate lipid-associated endotypes described in the lipid metabolic coding framework provide one testable example, but their value will depend on whether they improve prediction of progression or treatment response beyond conventional clinical variables.

### Spatial multi-omics and longitudinal disease mapping

Cross-sectional analysis of end-stage surgical tissue remains a major limitation in OA research because it cannot reliably distinguish initiating mechanisms from secondary responses. Spatial transcriptomics and single-cell profiling have revealed substantial cellular heterogeneity within cartilage and synovium [67–69, 71], but most current datasets do not simultaneously measure lipid abundance, metabolic flux, organelle dysfunction, or PTM states.

Future studies should therefore integrate spatial transcriptomics with spatial lipidomics, proteomics, PTM profiling, metabolic tracing, and quantitative imaging. Longitudinal sampling of synovial fluid, blood, imaging biomarkers, and, where feasible, joint tissues will be particularly important for distinguishing early adaptive responses from persistent programs that drive disease. Ultimately, a multidimensional OA atlas linking molecular states to anatomical location, disease stage, symptoms, and clinical outcomes could provide the foundation for mechanism-based patient stratification.

### Disease stage, sex, comorbidity, and human–model translation

OA mechanisms are likely to vary substantially across early, intermediate, and advanced disease. A pathway that is compensatory during early stress may become maladaptive when chronically activated, making disease stage an essential variable in both mechanistic studies and therapeutic development.

Sex and systemic comorbidities should also be incorporated more systematically. Obesity, diabetes, dyslipidaemia, ageing, and inflammatory comorbidity may alter the relative importance of metabolic, inflammatory, and biomechanical pathways. In particular, whether sex modifies joint lipid processing and metabolic responses remains insufficiently defined and warrants direct investigation.

Human translation remains another major challenge. Rodent models are indispensable for causal perturbation but differ from human OA in cartilage thickness, loading, ageing, and disease duration [156]. Greater use of human explants, longitudinal cohorts, organoid or tissue-engineered systems, and multimodal clinical samples will be needed to determine which experimental mechanisms are genuinely relevant to human disease.

### Precision intervention and next-generation clinical trials

Future OA trials should move beyond broad enrollment based primarily on radiographic severity. Biomarker-defined recruitment, disease-stage selection, and mechanism-linked outcome measures may increase the likelihood of detecting therapeutic benefit in biologically responsive subgroups.

Precision treatment will also require improved delivery. Cartilage is poorly vascularized and difficult to target, making intra-articular formulations, cartilage-penetrating carriers, and tissue-selective modulators particularly attractive [186, 189–192].

Ultimately, the central challenge is whether pathological molecular programs can be identified and therapeutically reset before irreversible joint failure occurs [187, 193–195]. For lipid-associated OA, this can be framed more specifically as a testable question: can the lipid code be read, and selectively reset, before cartilage degeneration becomes fixed?

### Validation gates from molecular discovery to clinical implementation

A precision OA framework requires sequential validation rather than a direct transition from molecular observation to treatment [188, 196, 197]. Initial findings must be reproducible across independent experimental systems and subsequently confirmed in human tissues and clinically relevant samples. Candidate biomarkers, particularly those obtainable through minimally invasive procedures, then need prospective evaluation to establish whether they can reliably identify distinct molecular states. Importantly, associations with disease severity alone are insufficient; these molecular signatures must also demonstrate an ability to predict treatment response. This would provide a stronger basis for randomized trials involving patients selected according to their biomarker profiles and disease stage. Such trials should assess not only structural outcomes but also their relationship to pain and physical function. Even when clinical efficacy is demonstrated, routine implementation will depend on standardized assays, acceptable safety, practical feasibility, cost-effectiveness, and consistent performance across healthcare settings [176, 179, 198–205]. This staged pathway is particularly important for the proposed lipid-associated endotypes, which currently remain hypothesis-generating rather than clinically actionable classifications.

## Conclusions

OA is a heterogeneous, multifactorial disease of the whole joint in which ageing, biomechanical stress, inflammation, metabolic dysfunction, and tissue-specific responses interact to drive progressive structural and functional deterioration. Understanding OA therefore requires integration of epidemiological patterns and risk factors with molecular events occurring across cartilage, synovium, subchondral bone, and the osteochondral interface.

Within this broader framework, lipid metabolic coding provides a mechanistic lens for understanding how lipid-derived substrates, metabolites, and stress signals may be differentially processed by joint cells and translated into persistent pathological states. Fatty-acid flux, de novo lipogenesis, lipid-droplet turnover, cholesterol handling, organelle stress, and lipid-sensitive post-translational modification networks illustrate how metabolic disturbances can influence chondrocyte identity, inflammatory activity, proteostasis, and inter-tissue communication. Importantly, these mechanisms should not be interpreted as uniformly causal or irreversible, and the proposed lipid-associated endotypes remain candidate, overlapping, and potentially stage-dependent biological programs rather than validated clinical categories.

Future progress will depend on longitudinal human studies that integrate molecular endotyping with spatial multi-omics, metabolic tracing, imaging, and clinically meaningful outcomes. Such approaches may help distinguish adaptive metabolic responses from disease-driving programs and identify patients most likely to benefit from mechanism-directed intervention. Ultimately, the goal is not to suppress lipid metabolism broadly, but to identify and selectively reset maladaptive pathways before joint damage becomes irreversible.

## Data Availability

Not applicable. This article is a narrative review and does not generate or analyse new datasets.
